# Antimicrobial resistance and associated risk factors in *Escherichia coli* isolated from Peruvian dogs: A focus on extended-spectrum β-lactamases and colistin

**DOI:** 10.14202/vetworld.2024.880-887

**Published:** 2024-04-20

**Authors:** Margot Ventura, Rosario Oporto-Llerena, Kathya Espinoza, Fernando Guibert, Antonio M. Quispe, Nidia Vilar, María López, Beatriz Rojo-Bezares, Yolanda Sáenz, Joaquim Ruiz, Maria J. Pons

**Affiliations:** 1 Grupo de Investigación en Dinámicas y Epidemiología de la Resistencia a Antimicrobianos - ”One Health”, Universidad Científica del Sur, Lima, Peru; 2 Facultad Biología, Universidad Ricardo Palma, Lima, Peru; 3 Universidad Continental, Huancayo, Peru; 4 Área de Microbiología Molecular, Centro de Investigación Biomédica de La Rioja (CIBIR), 26006 Logroño, Spain

**Keywords:** antibiotic resistance, colistin, dogs, extended-spectrum β-lactamases, Peru, risk factor

## Abstract

**Background and Aim:**

Established antimicrobial resistance (AMR) surveillance in companion animals is lacking, particularly in low-middle-income countries. The aim of this study was to analyze AMR and its risk factors in *Escherichia coli* isolated from dogs at two veterinary centers in Lima (Peru).

**Materials and Methods:**

Ninety dogs were included in the study. Antimicrobial susceptibility was established by disk diffusion, whereas microdilution was used to determine colistin susceptibility. Mechanisms related to extended-spectrum β-lactamases (ESBL) and colistin resistance were determined by polymerase chain reaction. Clonal relationships of colistin-resistant isolates were assessed by *Xba*I-pulsed-field gel electrophoresis.

**Results:**

Thirty-five *E. coli* strains were isolated. High levels of resistance to ampicillin (57.1%), nalidixic acid (54.3%), tetracycline (48.6%), and azithromycin (25.7%) were detected. Cephalosporin resistance levels were ≥20% and those for colistin were 14.3%. Twelve (34.2%) isolates were ESBL producers; of these, six *bla*_CTX-M-55_ (50.0%), 2 (16.6%) *bla*_CTX-M-15_, and 2 (16.6%) *bla*_CTX-M-8-like_ genes were found. The five colistin-resistant isolates were clonally unrelated, with four of them presenting amino acid codon substitutions in the *mgrB* gene (V8A) or mutations in the *mgrB* promoter (a12g, g98t, and c89t). Furthermore, dog age, <6 years (p = 0.027) and raw diet (p = 0.054) were associated with resistance to a greater number of antibiotic families.

**Conclusion:**

Despite small number of samples included, the study found that dogs studied were carriers of multidrug-resistant *E. coli*, including last-resort antimicrobials, representing a public health problem due to close contact between dogs and humans. This issue suggests the need for larger studies addressed to design strategies to prevent the spread of resistant micro-organisms in small animal clinics and domestic settings,

## Introduction

Antimicrobial resistance (AMR) is a significant public health threat worldwide, highlighting the health crisis linked to the worldwide increase in infections caused by pathogens resistant to last-line antibiotics [1]. In this sense, companion animals are often exposed to antibiotics due to the treatment of infections or prophylaxis [2] or through the consumption of food products [3], increasing selective pressure and leading to the development of multidrug resistance in their commensal bacteria [4]. Thus, pets can be reservoirs and hosts for resistant bacteria, raising concerns about the risks to both human and animal health [5].

*Escherichia coli* is a common member of the intestinal microbiota of humans and companion animals and is involved in many intestinal and extraintestinal infections [6, 7]. Because of its ubiquity and high facility for acquiring AMR genes, it is a good marker of antibiotic resistance [6–8]. In recent years, the emergence and rapid dissemination of *E. coli* producers of extended-spectrum β-lactamases (ESBL) [9, 10], carbapenemases [11, 12], and even the harboring of genes of resistance to last-resort antibiotics such as colistin [13] have become a paramount concern for animal health.

While a great variety of ESBLs have been described in the literature, the most widely distributed genes conferring resistance to broad-spectrum cephalosporins are the *bla*_CTX-M_ genes. Among these, *bla*_CTX-M-15_ is the most common ESBL in most regions of the world, whereas others, such as *bla*_CTX-M-27_ and *bla*_CTX-M-55_, are particularly relevant in specific areas [14]. Furthermore, regarding carbapenemases, reports of *bla*_NDM_, *bla*_KPC_, and *bla*_OXA-48_ have increased among enterobacterial in recent years, with this increase also being reported in veterinary microorganisms [15, 16]. Finally, regarding colistin resistance mechanisms, plasmid-mediated colistin resistance *mcr* genes and mutations in chromosomal genes have been frequently reported [17–19].

In small animals, antibiotic resistance and its associated factors have important implications for veterinary and public health [20]. However, scientific literature on this critical problem is scarce. In Peru, studies on the frequency of antibiotic-resistant microorganisms and their risk factors in companion animals are limited [8]. Thus, the aim of this study was to determine AMR levels and its risk factors in dogs from veterinary clinics in Lima, Peru.

## Materials and Methods

### Ethical approval and Informed consent

This study was approved by the Institutional Ethics Committee in Research with Animals and Biodiversity of the Universidad Científica del Sur (452-2020-POS99). In addition, all the owners of the canine companion animals provided written informed consent for sampling their animals.

### Study period and location

A cross-sectional study was conducted from January to July 2021 on clinically healthy dogs (i.e. Dogs attending for bathing or vaccination services) at two veterinary clinics in districts with different socioeconomic characteristics (Surco and Villa El Salvador) in Lima, Peru. Surco is one of the largest high-income residential districts of Lima (population ~400,000), whereas Villa El Salvador is one of the largest low-income residential districts of Lima (population ~2,400,000). All dogs that had consumed antibiotics within the 3 months before sampling were excluded from the study.

### Sampling

Convenience sampling was performed. Rectal swab samples were placed in Cary Blair preservation and maintained at 4–8°C during transportation to the Universidad Científica del Sur laboratories according to standardized protocols [5].

### Epidemiologic information

The owners provided epidemiological information on the canine companion animals. The canine data collected included sex, age (classified into two groups: <6 years and ≥6 years), diet (raw meat diet/prepared dry diet/mixed/home diet), owner’s history of antimicrobial use, and contact with other companion animals (yes/no).

### *E. coli* isolation

Samples were homogenized and seeded on MacConkey plates (Oxoid, Basingstoke, UK) or in McConkey agar containing disks of ceftazidime (30 μg) at one end of the plate and incubated at 37°C for 24 h. Then, isolated colonies compatible with *E. coli* growing within the ceftazidime-treated halos or without antibiotic pressure were selected and confirmed by polymerase chain reaction (PCR) amplifying the *E. coli*-specific *uidA* gene [21].

### Antimicrobial susceptibility

Antimicrobial susceptibility to ampicillin (10 μg), amoxicillin plus clavulanic acid (30 μg), ceftriaxone (30 μg), cefotaxime (30 μg), cefepime (30 μg), aztreonam (30 μg), trimethoprim-sulfamethoxazole (25 μg), nitrofurantoin (300 μg), tetracycline (30 μg), gentamicin (10 μg), nalidixic acid (30 μg), levofloxacin (5 μg), norfloxacin (10 μg), and meropenem (10 μg) was determined by the disk (Oxoid) diffusion method according to the clinical laboratory standard institute guidelines (CLSI) [22]. For azithromycin, a halo of ≤12 mm was considered to classify the isolates as resistant [23]. *E. coli* isolates were classified as multi-drug resistant (MDR) according to the classification of Magiorakos *et al*. [24] when resistant to at least three or more antibiotic families. To establish the presence of ESBLs, disks of amoxicillin plus clavulanic acid, cefotaxime, and ceftazidime were disposed as reported previously [22]. In addition, colistin susceptibility was determined using the microdilution technique following the recommendations of CLSI [22] and the European Committee on Antimicrobial Susceptibility Testing [23]. When more than one *E. coli* was isolated from the same dog, only one isolate was included in the study, except when the AMR patterns differed.

### Antibiotic resistance genes

In ESBL-positive phenotype isolates, the presence of *bla*_CTX-M_ (including *bla*_CTX-M1_, *bla*_CTX-M2_, *bla*_CTX-M8_, and *bla*_CTX-M9_) and *bla*_SHV_ genes was determined by PCR [25]. The amplified products were recovered and purified using the EZNA Gel Extraction Kit (Omega Bio Tek, Norcross, GA) and sequenced (Macrogen, Seoul, South Korea) to determine the specific gene. Meanwhile, the genes involved in carbapenem resistance were amplified by PCR: *bla*_VIM_, *bla*_IMP_*, bla*_KPC_*, bla*_NDM_, and *bla*_OXA-48_ [26]. In the case of colistin-resistant isolates, the presence of transferable *mcr-1* to *mcr-5* genes was analyzed by PCR [27]. In addition, the presence of point mutations in chromosomal genes, such as *mgrB*, was assessed by PCR [28] and subsequent sequencing (Macrogen, Seoul, South Korea).

### Phylogenetic and clonal analyses

The phylogenetic groups of the colistin-resistant isolates were established according to the Clermont extended scheme [29]. Clonal relationships among the colistin-resistant isolates were determined by pulsed-field gel electrophoresis (PFGE) of the total DNA digested with 40 U of *Xba*I restriction enzyme, as previously described by Gautom [30]. DNA fragments were resolved in 1 % agarose gels using the CHEF-DRIII system (Bio-Rad Laboratories Inc, Hercules, USA). The PFGE conditions used were at 6 V cm^-2^ at 14°C and with a pulse time ranging from 1 s to 30 s for 19 h. Normalization and processing of the PFGE images and construction of the dendrogram were performed using GelJ v2.0 software (https://sourceforge.net/projects/gelj/) [31]. A UPGMA (unweighted pair group method with arithmetic mean) tree was constructed using Dice similarity indices, complete linkage, 1% optimization, and 1.3 % position tolerance. Isolates were considered clonally related with a Dice similarity index ≥80% [32].

### Statistical analysis

A descriptive analysis was performed by summarizing the qualitative variables according to their absolute and relative frequencies and the quantitative variables according to their mean and standard deviation. During this analysis, intermediate-resistant phenotypes were considered to be resistant. In addition, AMR-associated factors were assessed by modeling the number of resistant antimicrobial families using a Poisson regression model. ESBL and multidrug resistance were also evaluated separately as secondary outcomes of interest. Finally, we estimated the prevalence ratio (PR) as the magnitude of the association of interest. During these analyses, diet categories included dry, raw meat, mixed, and homemade; however, for the regression analysis, we converted this variable to a dichotomous variable (raw vs. no raw diet). All statistical analyses were performed using the STATA MP v14.0 statistical package (Stata Corp LP, College Station, Texas), and each estimate was reported with their respective 95% confidence intervals (CI).

## Results

### Study population

Fecal samples from 90 dogs attending two veterinary clinics from different districts of Lima (Peru) were analyzed: 46 from Villa El Salvador (51.1%) and 44 from Surco (48.9%) (Table-1). Most samples were collected from female dogs (73.3%), those aged 6 years or older (51.1%), and dogs that lived with other pets (87.8%). Most dog diets were based on prepared dry food (52.2%), followed by raw meat (25.6%), mixed food (18.9%), and home food (3.3%). Regarding the owner’s history of antimicrobial use, 44.4% of the animal owners had consumed antibiotics within the last month.

**Table-1:** Epidemiological characteristics of the sampled dogs (total) and of those *Escherichia coli* positive.

Characteristics	*Escherichia coli*		Total (n = 90)

	Negative (n = 55)	Positive (n = 35)	
Districts			
Villa El Salvador district	28 (50.9)	18 (51.4)	46 (51.1)
Surco district	27 (49.1)	17 (48.6)	44 (48.8)
Age			
Age≥6 years	30 (54.6)	16 (45.7)	46 (51.1)
Age<6 years	25 (45.5)	19 (54.3)	44 (48.8)
Sex			
Female	41 (74.6)	25 (71.4.)	66 (73.3)
Male	14 (25.4)	10 (28.6)	24 (26.7)
Diet			
Dry	29 (52.7)	18 (51.4)	47 (52.2)
Raw meat	16 (29.1)	7 (20.0)	23 (25.6)
Mixed	7 (12.7)	10 (28.6)	17 (18.9)
Home	3 (5.5)	0 (0.0)	3 (3.3)
Lives together with other pets	49 (89.1)	30 (85.7)	79 (87.8)
The owner consumed antibiotics in the last month	17 (30.9)*	23 (65.7)*	40 (44.4)

* p = 0.001

### *E. coli* isolation and identification

Of the 90 fecal analyzed samples, 35 (38.9%) were *E. coli*-positive (of these, eight strains were isolated with the presence of CAZ and 27 from the medium without antibiotic disk). The characteristics of the dogs are shown in Table-1. When comparing the characteristics of the animals with *E. coli*-positive versus *E. coli*-negative fecal samples, there was a significantly frequent history of owners who had consumed antibiotics in the last month among the samples positive for *E. coli* (65.7% vs. 30.9%, p = 0.001) (Table-1).

### Antibiotic susceptibility

**Figure-1:**
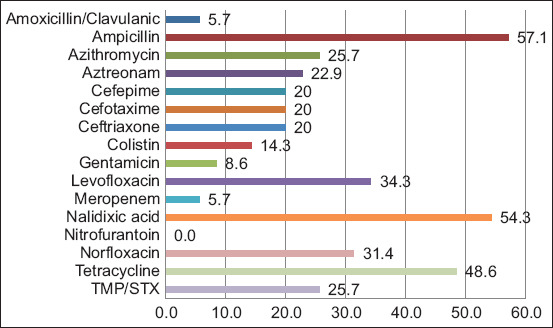
Percentage of resistance to different antibiotics in the 35 *Escherichia coli* isolates analyzed. (TMP/SXT=Trimethoprim-sulfamethoxazole).

The isolates showed high levels of resistance to antibiotics and were susceptible only to nitrofurantoin. The highest levels of resistance were to ampicillin (57.1%), nalidixic acid (54.3%), tetracycline (48.6%), levofloxacin (34.3%), and norfloxacin (31.4%). Resistance levels to cephalosporins were >20%, and the ESBL phenotype was detected in 12 isolates (34.3%). Meanwhile, colistin resistance reached 14.3%, with all five colistin-resistant isolates presenting a minimum inhibitory concentration (MIC) of 4 μg/mL (Figure-1). Twenty (57.1%) isolates were classified as MDR.

Regarding antibiotic resistance patterns, 3 (8.57%) isolates showed resistance to seven antimicrobial agents of different groups, 5 (14.28%) isolates showed resistance to six antimicrobial agents of different groups, and 3 (8.57%) isolates showed resistance to four and three antimicrobials, respectively. In addition, 1 (2.86%) isolate presented resistance to 2 antimicrobial agent groups and 5 (14.28%) isolates to one antimicrobial group. Note that 4 (11.4%) isolates did not present resistance to any antimicrobial agent (Supplementary Table-S1).

**Table-S1:** Antibiotic resistance patterns observed in the *Escherichia coli* isolates from fecal samples of dogs.

No. of Antibiotics	Antibiotic pattern	No. of isolates
1	AMP	4
	AZM	2
	Q	2
	CFS	1
	COL	1
2	TET, AZM	1
3	Q, AMP, AZM	2
	COL, TET, AZM	1
	TET, AMP, AZM	1
4	TET, Q, AMP, AZM	2
	COL, SXT, AMP, AZM	1
	CFS, ATM, TET, Q	1
6	AMC, CFS, ATM, TET, AMP, AZM	2
	CFS, ATM, SXT, TET, GEN, Q	2
	CFS, ATM, SXT, TET, Q, AMP	2
	CFS, ATM, SXT, TET, Q, AZM	2
	COL, CFS, ATM, SXT, Q, AMP	1
7	AMC, CFS, ATM, TET, Q, AMP, AZM	1
	CFS, ATM, SXT, TET, Q, AMP, AZM	1
	CFS, ATM, SXT, TET, GEN, Q, AMP	1

AMP = Ampicillin, AZM = Azithromycin, Q = Quinolones, CFS = Cephalosporines, COL = Colistin, TET = Tetracycline, SXT = Sulfamethoxazole/trimethoprim, ATM = Aztreonam, GEN = Gentamicin

### ESBL characterization

Twelve ESBL-producing *E. coli* isolates were detected (13.3% of the tested samples and 34.3% of the *E. coli*-positive samples). The *bla*_CTX-M-1G_ gene was amplified in 8 (66.7%) individuals, whereas no *bla*_SHV_ was detected. The variant *bla*_CTX-M-55_ was detected in six isolates, and *bla*_CTX-M-15_ was identified in the remaining two isolates. In addition, in two isolates *bla*_CTX-M-8G_ was amplified, and three isolates were positive for universal *bla*_CTX-M_, but no variant was determined. Finally, none of the ESBL-encoding genes sought were amplified in the two remaining isolates.

### Carbapenemase characterization

All isolates presented an intermediate phenotype to carbapenems. No positive results were detected among the carbapenemase genes assessed.

### Colistin characterization

**Figure-2:**
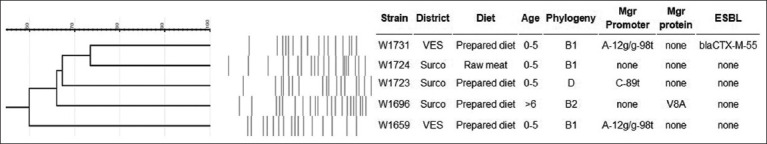
Clonal relationship and phylogeny of colistin-resistant isolates.

Five isolates were colistin-resistant, and mutations in the *mgrB* gene were found in four isolates. Three of these isolates presented alterations in the promoter region, including two possessing the base changes a-12→g and g-98→t and the remaining isolate showing the change c-89→t. Meanwhile, one isolate presented an amino acid codon substitution V8A (Figure-2). In addition, no *mcr* genes were detected.

### Phylogeny and clonal relationship between colistin-resistant isolates

The five colistin-resistant *E. coli* isolates were clonally unrelated. Three isolates belonged to group B1, and the other two belonged to groups D and B2, respectively (Figure-2).

### Factors associated with AMR

Regression analysis showed that animals aged 6 years presented *E. coli* isolates with resistance to a lower number of antimicrobial families (PR = 0.41; 95% CI: 0.19–0.90; p = 0.027). Although marginally significant (p = 0.054), dogs consuming raw food presented *E. coli* resistant to a higher number of antimicrobial families. The study did not have sufficient power to perform a multivariate Poisson regression model; likewise, no factor associated with ESBL or multidrug resistance was identified (Table-2).

**Table-2:** Factors associated with antimicrobial family resistance.

Associated factors	PR (95% CI)	p-value
Age		
<6 years	Ref	
≥6 years	0.41 (0.19–0.90)	0.027*
Raw diet		
No	Ref.	
Yes	1.78 (0.99–3.20)	0.054
Owner consumes antibiotics		
No	Ref.	
Yes	0.64 (0.35–1.19)	0.159
District		
Surco	Ref.	
Villa El Salvador	0.67 (0.36–1.25)	0.212
Sex		
Female	Ref.	
Male	0.86 (0.43–1.72)	0.678
Coexists with other animals		
No	Ref.	
Yes	0.96 (0.49–1.89)	0.912

PR = Prevalence ratio,

* p < 0.05

## Discussion

The present results demonstrate that companion animals, such as dogs, have antimicrobial-resistant *E. coli* that may be transmitted between animals and humans due to direct or indirect contact. Significantly high levels of antibiotic resistance were observed in canine *E. coli* isolates, with over half of the isolates being resistant to ampicillin and over half resistant to nalidixic acid and tetracycline. Moreover, approximately 40% of the isolates were cephalosporine-resistant and levofloxacin. Resistance levels to these antibiotics are important because these drugs are mainly used in human and veterinary medicine. Furthermore, this study highlights the presence of *E. coli* resistant to carbapenems and polymyxins, which is of concern because these antibiotic families are considered last resort antimicrobial treatment in human infections. In addition, we observed an association between dog age and the type of diet and the presence of *E. coli* resistance to a greater number of antibiotic families.

Previous studies characterizing resistance levels and mechanisms in *E. coli* recovered from companion animals in Peru are scarce [8]. However, studies conducted in Cajamarca (Northern Peru) [33] showed resistance levels of 61% for streptomycin and 51% for ampicillin, the antibiotics most commonly used in daily clinical practice in the region. The levels of antibiotic resistance in the present study were similar to those reported from Minas Gerais, a populous state in Brazil [3], although they did not report colistin resistance.

Our results are consistent with the increase in ESBL-producing *E. coli* reported in dogs worldwide, mainly related to the high use of β-lactams in clinical practice in veterinary medicine [34, 35]. The percentage of samples possessing ESBL-producing *E. coli* was 13.3%, similar to other countries in the region, such as Ecuador [36]. In the comparison among isolates, we found 34.3% of ESBL producers, which is a lower percentage than that reported in Thailand [10].

Regarding CTX-M variants, various ESBLs were found in the study, with *bla*_CTX-M-55_ being the most frequent. In addition, CTX-M-15 and CTX-M-8G were found. It is of note that CTX-M-55 has been previously reported in Peru in studies including samples of diverse origins (healthy human and clinical samples, marketed food, drinking water, and animals including wild mammals, poultry, livestock, and companion animals) [37–39]. Moreover, in ESBL studies in this region, CTX-M-55 has been the most frequently reported [3]. It should also be noted that CTX-M-15, the dominant variant worldwide [40], was not very frequently identified in this study. Similarly, other studies in the area have described the same finding, suggesting that CTX-M-15 is not the predominant ESBL in Peru [37, 41]. CTX-M-8G, a rare ESBL group, has been previously detected in dog feces in Northern Peru [38]. It is worth mentioning that some of the ESBL-positive strains did not amplify CTX-M, suggesting that other mechanisms may be involved, such as TEM or other less frequent ESBLs, a scenario that has already been reported in other cases in the country [25].

Colistin is used as a last resort for human clinical use against multiresistant bacteria, and it has not been marketed for veterinary use in small animals in the country since 2019 [42]. However, the results of our study showed that 14.3% of *E. coli* isolates were resistant to colistin, even in *E. coli* isolated from animals considered to be clinically healthy. In previous studies, *mcr*-1 carriage was associated with colistin resistance in *E. coli* from dogs and their owners [35]; however, resistance to colistin in the present study was probably related to chromosomal mutations, which were reported as a major mechanism in other Enterobacteria such as *Klebsiella pneumoniae* [43]. In this sense, three of five colistin-resistant isolates possessed mutations in the promoter region of *mgrB*, and another isolate possessed the amino acid substitution V8A. While no data about the effect of mutations in the promoter region of *mgrB* have been found in the literature, alterations in the promoter region of genes have the potential to affect (increasing, decreasing, or avoiding) the final expression levels of subsequently encoded peptides [44]. However, while the role of these alterations in promoter regions in final colistin MIC levels cannot be ruled out, they are outside the PhoP box and do not affect the -10 region or transcription start sites. Nevertheless, further studies are necessary to elucidate the effects of these alterations [45]. Meanwhile, the amino acid substitution V8A has been described in both colistin-resistant and colistin-susceptible isolates, suggesting a polymorphism or little effect on final colistin MIC levels [46]. Furthermore, PFGE and phylogenetic analysis demonstrated that these strains were clonally unrelated. Thus, exposure to colistin, which has been used in the avian sector for many years as a possible source of exposure through the food chain, can be considered [47].

When exploring the factors associated with AMR, we observed that diet appears to be a critical factor in resistance to certain families of antimicrobials. Although a dry diet based on different types of proteins, carbohydrates, grains, and other components that undergo dehydration was reported to be consumed by most of the dogs in our study, we found an association between consumption of a raw diet and resistance to a higher number of antimicrobial families. Raw food as the basis of a dog diet has gained interest recently because of studies or cases of cardiac pathologies related to different types of preservatives or ingredients used in dry foods [48]. However, the consumption of raw meat has been associated with a high risk of antibiotic-resistant *E. coli* and other food-borne bacteria, such as *Salmonella* [49]. It has also been associated with MDR *E. coli* in dogs with a raw meat-based diet in Brazil [3], emphasizing the importance of surveillance and control of this type of food, the consumption of which has increased in recent years. Moreover, this study found younger age to be a risk factor for AMR, despite age being related to longer exposure time to antimicrobials [50]. This fact could be related to the use of antimicrobials for treating diarrhea associated with dietary changes in puppies.

Close contact between pets, their owners, and the environment has led to the sharing of bacteria, including resistant bacteria [51]. Thus, the spread of bacteria from humans to animals and vice versa poses a risk for treating non-human infections and may compromise the treatment of infections in veterinary medicine. Therefore, the high consumption of antimicrobials by animal owners is noteworthy in our study, with more than 40% of the owners having consumed antibiotics in the previous month.

### Limitation

One of the limitations of this study was the lack of data related to the clinical history of the dogs from which the samples were collected, thereby precluding the analysis of the association between antibiotic resistance and underlying pathologies. Another limitation was that while the study power was sufficient to recognize the strongest associated factors, it was not sufficient to adjust a multivariate regression model.

## Conclusion

Our study demonstrates that dogs can be an important source of antibiotic resistance genes, including ESBLs and those involved in resistance to antibiotics of last resort, such as colistin. This is likely related to the high veterinary and dietary use of antibiotics, despite the prohibition of their use. The results of this study provide a better understanding of the presence of antibiotic-resistant bacteria in companion animals. They also highlight the need for strategies to prevent the spread of resistant strains in small animal clinics and domestic settings.

## Data Availability

The datasets generated during the current study are available from the corresponding author upon a reasonable request.

## Authors’ Contributions

MJP, JR, and MV: Conceptualization. MV, RO, KE, FG, AMQ, NV, ML, BRB, and YS: Acquisition of data, statistical analysis, and interpretation of data. All authors participated in drafting and reviewing of the manuscript. All authors have read, reviewed, and approved the final manuscript.
